# Donor-recipient risk assessment in extended criteria liver transplantation: a comparative analysis of prognostic scores

**DOI:** 10.31744/einstein_journal/2026AO2500

**Published:** 2026-08-12

**Authors:** Isadora Chen Rabello da Motta, Giovana Garcia Rossi, Fernanda Marques, Amanda Pinter Carvalheiro da Silva Boteon, Yuri Longatto Boteon

**Affiliations:** 1 Hospital Israelita Albert Einstein Faculdade Israelita de Ciências da Saúde Albert Einstein São Paulo SP Brazil Faculdade Israelita de Ciências da Saúde Albert Einstein, Hospital Israelita Albert Einstein, São Paulo, SP, Brazil.; 2 Hospital Israelita Albert Einstein São Paulo SP Brazil Hospital Israelita Albert Einstein, São Paulo, SP, Brazil.

**Keywords:** Liver transplantation, Tissue donors, Tissue and organ procurement, Prognosis

## Abstract

**Objective:**

This study aimed to evaluate the association between the use of extended criteria donors and post-transplant outcomes in a Brazilian liver transplantation center and to compare the prognostic performance of established severity scores.

**Methods:**

We conducted a retrospective observational study of liver transplantation performed between 2016 and 2020. Patient and graft survivals were assessed at 3, 12, 36, and 60 months. Extended criteria donor status was defined according to the Eurotransplant criteria. The predictive values of the Donor-Model for End-Stage Liver Disease, Donor Risk Index, and Balance of Risk scores were analyzed using categorical and continuous models.

**Results:**

Among 414 transplants, 62% (n=258) involved extended criteria donor grafts. One- and five-year patient survival rates were 89.5% and 82.5%, respectively, with no significant difference between extended criteria donor and non-extended criteria donor groups (log-rank p=0.074). The extended criteria donor classification was not independently associated with mortality. The Balance of Risk score was the only prognostic model significantly associated with mortality and graft loss. Each one-point increase in the Balance of Risk was associated with a 7.3% increase in mortality risk (hazard ratio [HR]=1.073; 95% confidence interval [95%CI], 1.016-1.133; p=0.012) and a 17.4% increase in graft loss risk (HR=1.174; 95%CI=1.073-1.284; p<0.001).

**Conclusion:**

In this cohort, extended criteria donor status alone did not predict inferior survival, whereas the Balance of Risk score demonstrated consistent prognostic discrimination. These findings support the use of integrated donor-recipient risk assessment to guide organ acceptance and allocation decisions in liver transplantation.

## Highlights

The 1- and 5-year survival rates in extended criteria donor group were 89.5% and 82.5%, respectively.

Eurotransplant extended criteria donor status alone was not correlated with inferior survival outcomes.

The BAR score was the only model associated with mortality and graft loss.

Each one-point BAR increased mortality by 7.3% and graft loss by 17.4.

## In Brief

Integrated donor-recipient risk assessment offers a more informative framework than extended criteria donor status alone for liver transplantation. The BAR score was the only model consistently associated with mortality and graft loss, which supports the use of composite severity scores to guide allocation and optimize outcomes.

## Graphical abstract

**Figure:**
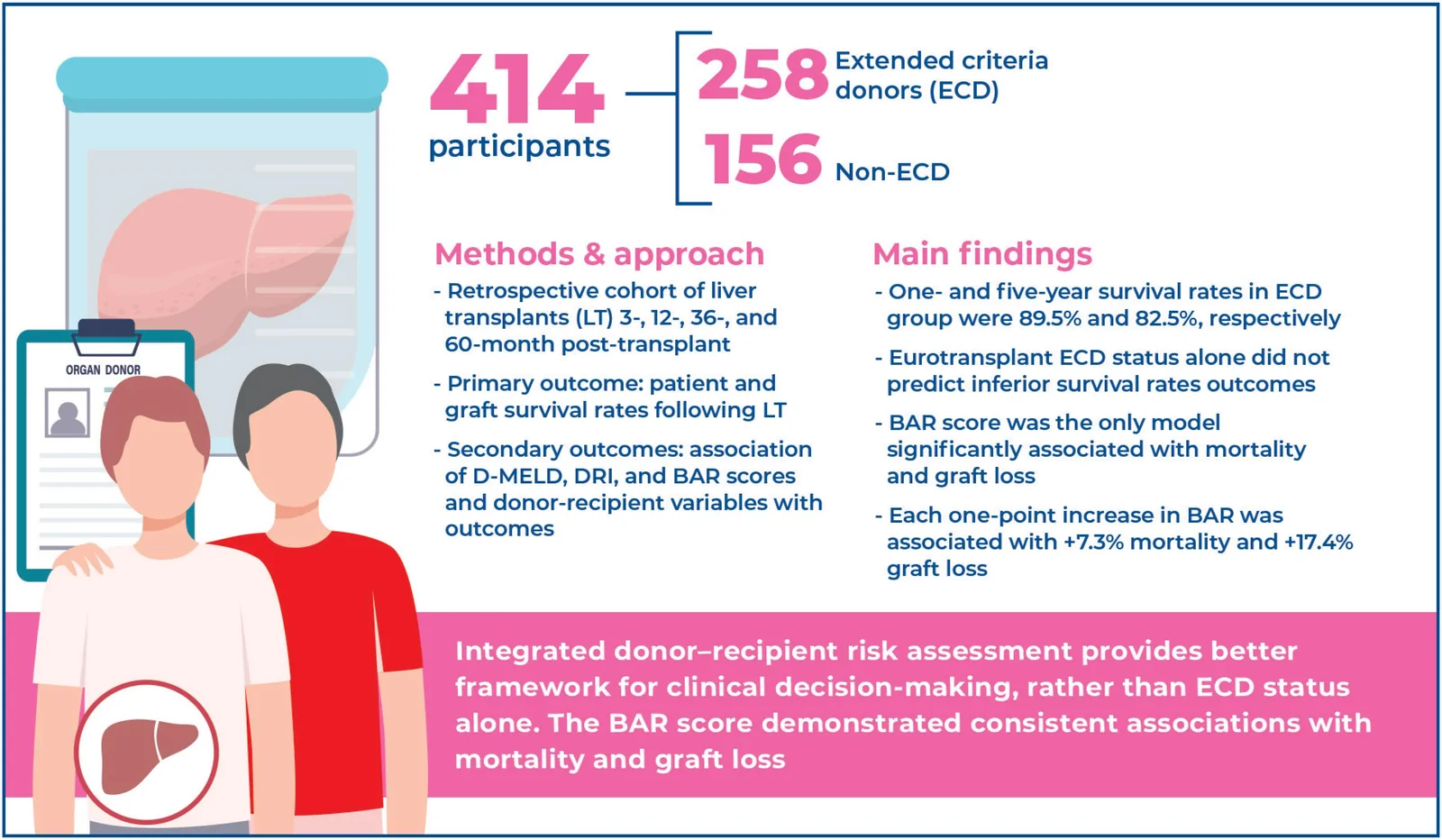

## INTRODUCTION

Liver transplantation (LT) is the standard therapy for end-stage liver disease; however, organ availability continues to limit access worldwide.^(1,2)^ To mitigate this shortage, LT programs have progressively broadened donor acceptance, increasingly relying on grafts that fall outside traditional "standard" profiles, commonly referred to as extended criteria donor (ECD) organs. Extended criteria donor status is assigned when donor characteristics associated with poorer graft performance are present, including higher rates of early allograft dysfunction and primary non-function.^(3,4)^ Despite the potential for increased perioperative risk, ECD grafts have become integral to contemporary LT activity.^(5)^

Extended criteria donor grafts are commonly used across allocation systems. In Brazil, Eurotransplant-based definitions have identified ECD criteria in a large proportion of donor offers,^(6)^ with similarly high rates reported in Eurotransplant member countries, North America, and other cohorts using comparable or alternative definitions.^(7-13)^ This widespread reliance underscores the need for structured approaches to quantify and manage the risk when marginal grafts are used.

As use of ECD increases, transplant teams require practical tools to support donor-recipient matching and anticipate post-transplant outcomes. Multiple prognostic models have been proposed to inform acceptance and matching decisions, particularly when donor risk features are present.^(14-16)^ The Balance of Risk (BAR) score may outperform commonly used alternatives such as the Model for End-Stage Liver Disease (MELD), Donor-MELD (D-MELD), and the Donor Risk Index (DRI) in predicting post-LT outcomes.^(16)^ By integrating donor and recipient variables into a single composite estimate, the BAR may be especially useful when evaluating higher-risk scenarios, including transplants performed with ECD grafts.^(16)^

## OBJECTIVE

Accordingly, we aimed to assess post-liver transplantation patient and graft survival by Eurotransplant-defined extended criteria donor status and evaluate whether BAR, D-MELD, and DRI can discriminate mortality and graft loss in this cohort.

## METHODS

### Study design and ethics

We performed a retrospective cohort study of consecutive LTs conducted from January 2016 to December 2020 at the liver transplant program of the *Hospital Israelita Albert Einstein*, São Paulo, Brazil. The study was approved by the Research Ethics Committee of *Hospital Israelita Albert Einstein* (CAAE: 54052121.4.0000.0071; # 5.197.892).

### Extended criteria donor definition

Extended criteria donor status was assigned when ≥1 Eurotransplant criterion was present:^(4)^ age >65 years; body mass index (BMI) >30kg/m^2^; intensive care unit (ICU) stay with mechanical ventilation for >7 days; hepatic steatosis >40%; total serum bilirubin (TB) >3mg/dL; serum sodium >165mmol/L; aspartate aminotransferase (AST) >105U/L; alanine aminotransferase (ALT) >105U/L.^(4)^

### Data collection

Clinical, demographic, and procedural data from the donors and recipients were obtained. Recipient variables included the presence of hepatocellular carcinoma (HCC), etiology of liver disease, and MELD score at the time of transplantation. Only the laboratory MELD score, based on objective biochemical parameters, was used; exception points granted for special indications (e.g., HCC or refractory ascites) were not considered. Information on the recipient's height and weight at registration and during waiting-listing was collected.

Donor variables included sex, age, race, geographic origin (local, regional, national), cause of death, ICU length of stay, orotracheal intubation duration, height, estimated weight, and laboratory values (serum sodium, TB, AST, and ALT). The procedure-related variables included cold and warm ischemia times, steatosis severity, and ischemia-reperfusion injury assessed through biopsy after reperfusion.

Donor BMI was derived from procurement-reported height and estimated weight; because deceased donor weight is often estimated in Brazil, BMI-based classification may be imprecise. Steatosis was recorded based on the macroscopic assessment of the retrieval team, as procurement biopsies were not routinely performed in this setting. Post-reperfusion biopsies were not included because of the potential influence of ischemia-reperfusion injury and the absence of standardized pre-reperfusion histological assessment in Brazilian transplant practice.

Prognostic scores were calculated for each transplant, including the MELD,^(14)^ DRI,^(15)^ BAR score^(16)^ and D-MELD. The D-MELD score was derived as the product of the recipient MELD score and donor age, and used as an indicator of graft failure risk by integrating recipient disease severity with donor age.^(14)^ DRI score summarizes donor-related factors such as age, cause of death, and cold ischemia time to estimate the likelihood of graft failure, with higher values reflecting higher risk.^(15)^ BAR score integrates both donor and recipient variables, including MELD score, donor age, retransplantation status, cold ischemia time, and need for life support, to predict post-transplant mortality.^(16)^

### Endpoints

The primary outcome was overall patient survival after LT, comparing ECD to standard donor grafts (without marginality criteria from the Eurotransplant Manual), assessed at 3-, 12-, 36-, and 60-month post-transplant. Survival curves were stratified based on the number of ECD criteria each patient possessed (0, 1, 2, or ≥3).

Graft survival was assessed in a similar manner, with censoring at the time of death or the last clinical follow-up. The association between donor characteristics (including marginal criteria) and death or retransplantation outcomes was analyzed.

To evaluate the prognostic value of the D-MELD, DRI, and BAR scores, we assessed their association with patient and graft outcomes using continuous and categorical models and compared them using ROC curve analysis.

### Statistical analysis

Statistical analyses were conducted using R statistical computing environment version 4.1.1. A significance level of 5% (p<0.05) was adopted. Continuous variables were summarized using means with standard deviations or medians, interquartile ranges, and maximum and minimum values. Categorical variables were reported using frequency distribution.

Associations between outcomes and categorical variables were evaluated using Fisher's exact test, whereas comparisons involving continuous variables were performed using the Mann-Whitney U test. Patient survival was estimated using the Kaplan-Meier method, with group differences determined using the log-rank test. Variables demonstrating significant differences in survival analyses were subsequently entered into Cox proportional hazards models to assess the independent effects of covariates on patient mortality and graft failure.

The distribution of continuous variables was assessed using the Shapiro-Wilk test. The statistical power for the survival analysis was estimated by fixing the group sizes and 1-year event probabilities. The event probabilities were 0.844 and 0.777 for the ECD and standard donor groups, respectively, with a 5% significance level. The minimum detectable hazard ratio (HR) for the available sample size without loss of statistical power was 1.40.

## RESULTS

### Recipient and donor data analysis

The most prevalent etiology of liver disease was hepatitis C virus infection (n=121; 29.23%), and 41.55% had HCC (n=172). The median MELD score was 18 (interquartile range [IQR], 13-25). The median D-MELD score was 763 (IQR, 470-1,085.5), DRI was 1.77 (IQR, 1.51-2.02), and BAR was 7 (IQR, 4-10). The median number of extended criteria in the sample was 1 (IQR, 0-2).

When analyzing intra-procedural variables, the median cold ischemia time was 400 min (IQR, 308.5-470), and the median warm ischemia time was 40 min (IQR, 35-45). The leading cause of donor death was a cerebrovascular accident (n=230; 55.56%). Of the donors, 62.3% (n=258) met the ECD criteria (i.e., ≥ 1 marginality criterion). Among ECD donors, 30.2% (n=125) possessed one criterion, 23.4% (n=97) had two, and 8.7% (n=36) had three or more. The primary nonfunction rate was 3.1% (n=13).

Table 1 presents the baseline demographic and clinical characteristics of LT recipients and donors, and table 2 summarizes the distribution of donor-recipient prognostic scores, including D-MELD, DRI, and BAR.

**Table 1:** Baseline clinical and demographic characteristics of liver transplant recipients and donors

Recipient variables		n (%)
Etiology		
	HCV	121 (29.23)
	Alcohol	100 (24.15)
	Crypto	63 (15.22)
	NASH	34 (8.21)
	Other	96 (23.19)
MELD		
	<10	36 (8.70)
	10-19	203 (49.03)
	20-29	109 (26.33)
	≥30	66 (15.94)
Donor variables		
Age >65 years, yes		25 (6.04)
Donor sex, male		236 (57.00)
Cause of death		
	Trauma	117 (28.26)
	CVA	230 (55.56)
	Anoxia	36 (8.70)
	Other	31 (7.49)
Donor Race		
	White	197 (47.58)
	Black	51 (12.32)
	Other	166 (40.10)
Location		
	Local	316 (76.33)
	Regional	85 (20.53)
	National	13 (3.14)
ICU >7 days, yes		114 (27.54)
BMI >30kg/m^2^, yes		47 (11.38)
Na >165mmol/L, yes		71 (17.15)
AST >90U/L, yes		106 (25.60)
ALT >105U/L, yes		66 (15.94)
TB >3mg/dL, yes		5 (1.21)

Crypto: cryptogenic cirrhosis; NASH: nonalcoholic steatohepatitis; HCV: hepatitis C virus; MELD: model for end-stage liver disease; ECD: extended criteria donor; ICU: intensive care unit; BMI: body mass index; Na: sodium; AST: aspartate transaminase; ALT: alanine transaminase; TB: total bilirubin.

**Table 2:** Distribution of donor-recipient prognostic scores in the liver transplant study population

Donor-recipient matching variables		n (%)
D-MELD (classified)		
	0-399	79 (19.13)
	400-799	142 (34.38)
	800-1,199	109 (26.39)
	1,200-1,599	46 (11.14)
	≥1,600	37 (8.96)
DRI (classified)		
	≤1.4	47 (11.35)
	>1.4 ≤1.8	173 (41.79)
	>1.8	194 (46.86)
BAR (classified)		
	0-4	132 (31.88)
	5-8	133 (32.13)
	9-13	111 (26.81)
	14-18	37 (8.94)

D-MELD: donor-model for end-stage liver disease; DRI: donor risk index; BAR: balance of risk core.

### Patient survival analysis

The estimated probabilities of overall patient survival after LT were 94.10%, 89.50%, 86.20%, and 82.50% at 3, 12, 36, and 60 months, respectively.

Figure 1A shows the Kaplan-Meier survival curves comparing recipients of ECD grafts with those receiving standard criteria donor (non-ECD) organs. Survival appeared higher among recipients of non-ECD grafts; however, the difference was not significant (log-rank p=0.074). Cox proportional hazards regression analysis was consistent with these findings, as no significant association between ECD status and mortality risk was observed (HR=0.65, 95% confidence interval [95%CI], 0.40-1.72, p=0.07]. Figure 1B presents the Kaplan-Meier curves stratified according to the number of ECD risk factors (0, 1, 2, or ≥3). No significant differences in survival were observed between the groups (log-rank p=0.36).

**Figure 1:**
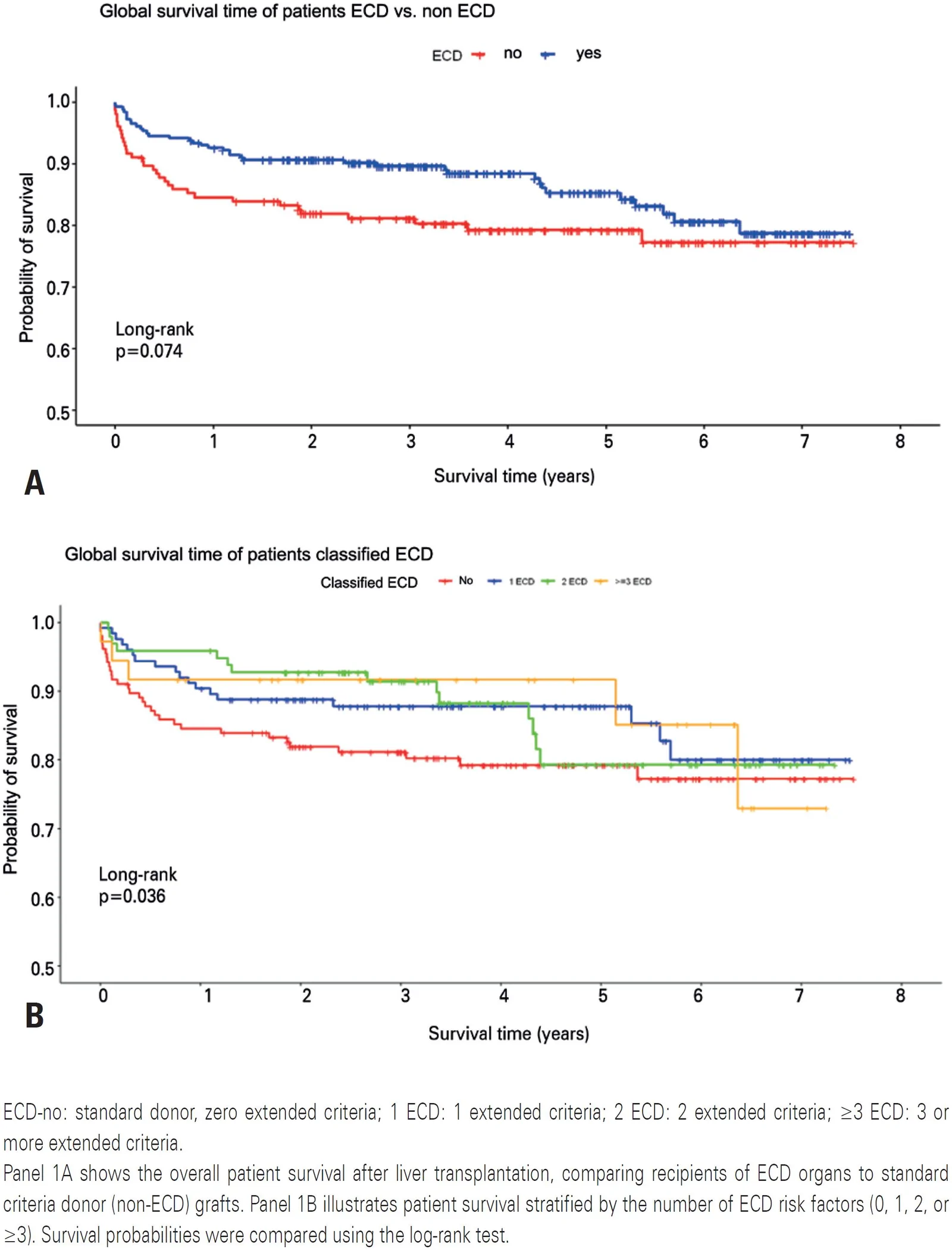
Kaplan-Meier survival analysis of liver transplant recipients according to extended criteria donor classification

### Severity score analysis

As shown in table 3, the BAR score was significantly associated with patient death (p=0.042) and graft loss (p<0.001). In contrast, the D-MELD and DRI scores showed no significant differences between the groups for either outcome.

**Table 3:** Association between donor-recipient prognostic scores and the risk of patient death and graft loss after liver transplantation

		(n)	Median [IQR]	p value
D MELD				
Death				0.169
	No	(345)	741 [462-1,064]	
	Yes	(69)	854 [560-1,110]	
Graft loss				0.298
	No	(389)	767 [468-1,064]	
	Yes	(25)	720 [532-1,408]	
	DRI			
Death				0.717
	No	(345)	1.77 [1.51-2.03]	
	Yes	(69)	1.75 [1.52-1.99]	
Graft loss				0.266
	No	(389)	1.77 [1,51-2,05]	
	Yes	(25)	1.75 [1,52-1,86]	
	BAR			
Death				0.042
	No	(345)	7 [3-10]	
	Yes	(69)	8 [4-11]	
Graft loss				<0.001
	No	(389)	7 [3-10]	
	Yes	(25)	10 [7-12]	

Significance was assessed using the Mann-Whitney U test. The bold p-values indicate significance.

D-MELD: donor-model for end-stage liver disease; DRI: donor risk index; BAR: balance of risk score; IQR: interquartile range (1^st^ and 3^rd^ quartiles)

A comparison of survival curves stratified by categorized prognostic scores (BAR, D-MELD, and DRI) demonstrated that only the BAR score was significantly associated with differences in patient survival (log-rank p<0.001), as shown in table 4. The D-MELD and DRI classifications did not show significant differences in survival.

**Table 4:** Association between categorized prognostic scores and patient or graft survival based on log-rank analysis

Variable	Test	Patient p value	Graft p value
BAR classified	Log-rank	<0.001	<0.001
D-MELD classified	Log-rank	0.31	0.36
DRI classified	Log-rank	0.78	0.51

Log-rank test p-values comparing survival distributions (Kaplan-Meier curves) for patient and graft outcomes according to the categorized donor-recipient prognostic score.

D-MELD: donor-model for end-stage liver disease; DRI: donor risk index; BAR: balance of risk score.

Figure 2 shows the Kaplan-Meier curves for patients stratified by BAR score categories (0-4, 5-8, 9-13, 14-18, and >18), revealing a clear distinction between the groups and suggesting the discriminatory capacity of the BAR score in predicting post-transplant survival.

**Figure 2:**
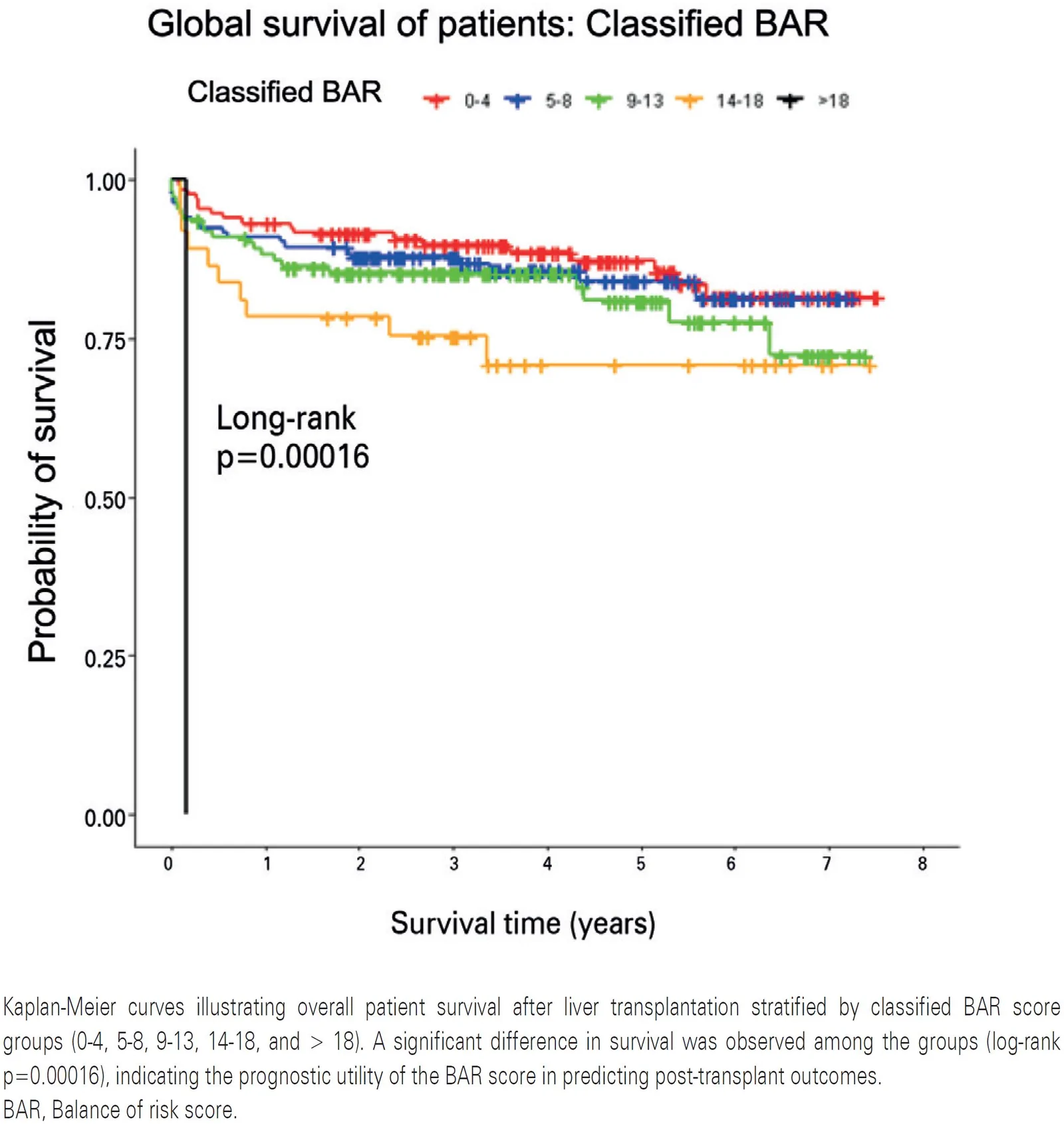
Kaplan-Meier survival analysis according to the classified Balance of Risk (BAR) score

Given this observed association, Cox proportional hazards regression was performed with the BAR score modeled as a continuous variable. The analysis showed a significant association between BAR score and mortality (HR=1.073, 95%CI=1.01-1.13, p=0.01), indicating that each one-point increase in the BAR score was associated with a 7.3% increase in the risk of death.

In the Cox regression analysis for graft loss, the BAR score was significantly associated with the risk (HR=1.174, 95%CI=1.07-1.28, p<0.001). Each one-point increase in the BAR score corresponded to a 17.4% higher risk of graft loss, supporting its prognostic relevance in LT.

## DISCUSSION

This study has two principal findings. First, the use of grafts meeting the Eurotransplant ECD definitions was not independently associated with inferior long-term survival in this cohort. Second, among the evaluated prognostic models, only the BAR score consistently demonstrated significant associations with mortality and graft loss, supporting its clinical utility as an integrated risk assessment tool for LT.

The absence of a significant survival disadvantage among ECD recipients reinforces the concept that donor marginality, when considered in isolation, may not fully determine post-transplant outcomes. The adoption of ECD grafts is a widely used response to persistent organ scarcity.^(17)^ As transplant programs increasingly accept donors with expanded criteria, the clinical question shifts from whether ECD grafts should be used to how their risks can be quantified and balanced appropriately. Our findings suggest that structured donor-recipient matching may mitigate the adverse effects traditionally attributed to ECD classification alone.

Despite frequent ECD use, the overall survival in our cohort was in line with the outcomes reported by high-volume programs,^(18)^ despite the substantial proportion of ECD graft utilization. This observation highlights that outcomes depend on a multifactorial interplay involving donor features, recipient severity, ischemia time, and perioperative management. Therefore, the binary categorization of grafts as ECD or non-ECD may oversimplify a complex biological and clinical reality.

The high prevalence of ECD use at our center mirrors national and international patterns. In Brazil, analyses applying Eurotransplant-based criteria have demonstrated ECD rates approaching 78%.^(6)^ Other Brazilian studies have similarly documented extensive reliance on ECDs, although with heterogeneous definitions.^(12,13,19)^ These data confirm that ECD transplantation is firmly integrated into routine practice rather than being an exceptional strategy. Consequently, robust tools to support decision-making are essential to ensure that the expanded utilization does not compromise acceptable outcomes.

International registry analyses further contextualized these findings. A large retrospective evaluation of the OPTN/UNOS database from 2001 to 2020 demonstrated significant improvement in graft survival among ECD recipients over time, with more recent ECD cohorts achieving outcomes that compared favorably to earlier non-ECD eras.^(20)^ Similarly, a study combining data from the German Transplant Registry and the United States Scientific Registry of Transplant Recipients (SRTR), encompassing over 90,000 donors and more than 70,000 adult transplants, reported that most Eurotransplant-defined ECD criteria were not independently associated with reduced short-term survival, except for donor age.^(21)^ These findings suggest that the prognostic impact of ECD characteristics is dynamic and influenced by evolving clinical expertise and allocation practices.

In this context, the performance of prognostic models is central. In our study, the BAR score was the only evaluated index significantly associated with mortality and graft loss, regardless of whether it was analyzed categorically or as a continuous variable. Each one-point increment in the BAR was associated with a 7.3% increase in mortality risk and a 17.4% increase in graft loss risk. In contrast, D-MELD and DRI were not significantly associated with outcomes in this cohort. These results underscore the potential advantages of composite scores that integrate donor and recipient variables, rather than focusing predominantly on a single domain.

The superiority of BAR over MELD-based or donor-focused indices has been previously suggested.^(16)^ A Brazilian retrospective study including 402 liver transplant recipients between 1997 and 2012 reported significantly lower 1-year survival among patients with BAR ≥11 compared to those with lower scores (44% *versus* 69%, p=0.001), although long-term predictive performance was considered limited.^(22)^ Our findings extend this evidence by demonstrating consistent associations between BAR and both mortality and graft loss in a contemporary cohort with follow-up extending to 5 years using internationally standardized ECD criteria.

Organ utilization and discard patterns provide an additional layer of interpretation. Brazilian data indicate that each additional ECD criterion increases the likelihood of organ refusal, with one analysis showing a 64.4% rise in refusal probability per added risk factor.^(6)^ Moreover, evaluations of graft discard in high-volume centers have revealed that many livers are declined due to perceived graft-related abnormalities.^(19)^ These patterns highlight the potential disconnect between risk perception and actual post-transplant performance. In this scenario, validated composite scores may help support more objective and reproducible decision-making during organ assessment.

This study had certain limitations. The retrospective and single-center design may restrict generalizability. Although the cohort included 414 transplants, it may not reflect the full heterogeneity of the national practice. Donor BMI was derived from estimated rather than directly measured weight, potentially introducing imprecision, and hepatic steatosis was assessed macroscopically rather than histologically. Additionally, allocation policies, regional donor availability, and healthcare disparities may have influenced the outcomes but were not directly modeled.

Despite these limitations, this study contributes relevant data to the discussion on ECD utilization and prognostic modeling after LT. By applying internationally recognized ECD definitions and directly comparing commonly used severity scores, we demonstrated that composite donor-recipient risk stratification, particularly using the BAR score, may provide a more nuanced and clinically useful framework than reliance on ECD classification alone.

## CONCLUSION

Grafts meeting Eurotransplant extended criteria definitions were not independently associated with inferior patient or graft survival in this cohort, suggesting that donor classification alone may not adequately determine post-transplant prognosis. Our findings indicate that integrated donor-recipient risk assessment provides a more informative framework for clinical decision-making, with the BAR score demonstrating consistent associations with both mortality and graft loss. These results support the incorporation of composite severity models into allocation and acceptance strategies to optimize graft utilization while maintaining favorable long-term outcomes.

## Data Availability

The underlying content is contained within the manuscript.
